# The first reported case of California encephalitis in more than 50 years.

**DOI:** 10.3201/eid0703.010316

**Published:** 2001

**Authors:** B F Eldridge, C Glaser, R E Pedrin, R E Chiles

**Affiliations:** University of California, Davis, California 95616, USA. bfeldridge@ucdavis.edu

## Abstract

A recent case of California encephalitis, a rare mosquito-borne viral disease, represents only the fourth ever reported and the first since the initial three cases in 1945. This case was diagnosed retrospectively on the basis of a rise in antibody titer between acute- and convalescent-phase serum samples.


### The First Reported Case of California Encephalitis in More Than 50 Years

**Bruce F. Eldridge,* Carol Glaser,† Robert E. Pedrin,‡ and Robert E. Chiles***
*University of California, Davis, California, USA; †California Department of Health Services, Berkeley, California, USA; and ‡Family Practice, Greenbrae, California, USA

A recent case of California encephalitis, a rare mosquito-borne viral disease, represents only the fourth ever reported and the first since the initial three cases in 1945. This case was diagnosed retrospectively on the basis of a rise in antibody titer between acute- and convalescent-phase serum samples.

The arbovirus California encephalitis virus was first isolated in 1943 from mosquitoes collected in Kern County, California (1). Two years later, three human cases of encephalitis were attributed to this new virus (2); all three cases were in residents of Kern County in the Central Valley of California. The best-documented case occurred in a 2-month-old Hispanic boy who had encephalitis and resultant developmental delay. There was strong laboratory evidence confirming infection from the presence of neutralizing antibodies to California encephalitis, but not to St. Louis encephalitis virus or western equine encephalomyelitis virus. Serum from a 7-year-old boy hospitalized with encephalitis also had neutralizing antibodies to California encephalitis. Serologic tests were inconclusive in a third possible case in a 22-year-old agricultural worker with mild encephalitis; neutralizing antibodies against both California encephalitis and St. Louis encephalitis were detected.

Since the original virus was isolated, other viruses have been isolated that are closely related to California encephalitis. This group of related viruses is now classified as the California serogroup, one of 16 serogroups within the genus *Bunyavirus*, family Bunyaviridae. Several other human pathogens (e.g., Jamestown Canyon virus, La Crosse virus, and Tahyna virus) also belong to the California serogroup. Little human disease was associated with these viruses until 1960, but now California serogroup virus infections are the most commonly reported cause of arboviral encephalitis in the United States. From 1996 to 1998, approximately three times as many reported human cases of arboviral encephalitis were caused by California serogroup viruses as were reported for western equine encephalomyelitis virus, St. Louis encephalitis, and eastern equine encephalomyelitis viruses combined (3). However, since the three original cases from California, no further cases of human disease caused by the prototype California encephalitis had been reported (4). Campbell et al. summarized results of surveys for human antibodies to California serogroup viruses in California (5).

Address for correspondence: Bruce F. Eldridge, Department of Entomology, University of California, Davis, CA 95616, USA; fax: 530-752-1537; e-mail: bfeldridge@ucdavis.edu

### Case Report

In June 1996, a 65-year-old man who lived in Marin County, California, became ill with blurred vision and dizziness. Eight days after the onset of symptoms, he visited his primary physician. A physical examination was remarkable only for nystagmus. Laboratory studies included leukocytes $8.2x10^3$, hematocrit 45.8%, and a chemistry panel that was normal except for phosphorus 4.5 mg/dL (normal range 2.7-4.4 mg/dL), cholesterol 265 mg/dL (normal range 125-200 mg/dL), and high-density lipoprotein cholesterol 67 mg/dL (normal range 35-60 mg/dL). A magnetic resonance image and arteriogram were normal. One month after the initial visit, the patient no longer complained of blurred vision or vertigo, and nystagmus had disappeared. Two years after the episode, he had no neurologic sequelae.

The patient lived in a suburban area of Marin County, approximately 1 km from a large brackish marsh bordering San Francisco Bay. He had traveled outside the United States the previous February, when he had visited Egypt and several Caribbean islands. He had not traveled outside California between this time and the onset of his illness in June, 4 months later.

An acute-phase serum specimen was sent to the Viral and Rickettsial Disease Laboratory, California Department of Health Services. Indirect immunofluorescence antibody tests were negative for St. Louis encephalitis and Western equine encephalomyelitis virus, as were serum samples taken 8 and 16 days after onset of illness. However, when this series of samples was tested by neutralization test 2 years later at the Arbovirus Research Unit of the University of California Center for Vector-Borne Disease Research as part of a retrospective study of arboviruses, the 8-day sample showed an antibody titer of 1:80 and the 16-day sample an antibody titer $\leq$ 1:320 to California encephalitis (Table).

California encephalitis-related arboviruses included in the tests were snowshoe hare, La Crosse, Jamestown Canyon, Morro Bay, and Tahyna. Tahyna, an important California serogroup virus widely distributed in Europe and Asia, was included because of the patient's travel history. Northway, Main Drain, and Lokern viruses, members of the Bunyamwera serogroup occurring in California, were also included, as were western equine encephalomyelitis virus and St. Louis encephalitis.

Table. Neutralizing antibody titers to 10 arboviruses in paired serum samples of a patient with neurologic symptoms, California, 1996

| Virus (strain) | Acute-phase serum[a] | Convalescent-phase serum[b] |
|---|---|---|
| California encephalitis (BFS-283) | 1:80 | 1:320 |
| Snowshoe hare (original) | 1:80 | 1:160 |
| La Crosse (prototype) | 1:40 | 1:40 |
| Jamestown Canyon (BFS 4474) | 1:20 | 1:20 |
| Morro Bay (DAV 457) | 1:80 | 1:40 |
| Tahyna (Bardos 92) | 1:40 | 1:20 |
| Northway (BFN 2654) | 1:20 | <1:20 |
| Main Drain (BFS 5015) | <1:20 | <1:20 |
| Lokern (BFS 5183) | <1:20 | <1:20 |
| Western equine encephalomyelitis (BFS 1703) | <1:20 | <1:20 |
| St. Louis encephalitis (BFS 1750) | <1:20 | <1:20 |

[a]Sample taken 8 days after onset of symptoms.
[b]Sample taken 16 days after onset of symptoms.

The sera were tested by plaque reduction-serum dilution neutralization with African green monkey kidney (Vero) cell cultures, according to the protocol of Campbell et al. (6). An increase in titer between the acute- and convalescent-phase samples was found only for California encephalitis and snowshoe hare virus. The titers for the convalescent-phase serum were 1:320 for California encephalitis and 1:180 for the closely related snowshoe hare virus (Table), which has never been reported in California. The patient had not traveled recently to alpine areas of the state, where snowshoe hare virus might be expected to occur.

### Conclusions

Because the patient's travel occurred several months before his illness, exposure to mosquitoes near his home is the most likely route of infection. The most common mosquito species in salt marshes in Marin County are *Aedes washinoi* and *Ae. squamiger* (7). Bloodsucking adult females of both these species are usually present in late winter to early spring. *Ae. dorsalis* also occurs in this area, but later in the year. *Culiseta inornata* occurs frequently near salt marshes. California encephalitis has not been recovered from any of these species in California; most isolates have come from *Ae. melanimon* in the Central Valley (8).

Campbell et al. (5) found that most seropositive samples from humans, mostly from high elevations (>1,000 m) in California, were apparently infected with Jamestown Canyon virus. However, these investigators also reported that approximately 35% of samples from horses in low elevations (<1,000 m) in California showed evidence of prior infection with California encephalitis (9), but they did not test samples from the San Francisco Bay area. Fulhorst et al. (10) reported that 57% of the horses sampled in Marin County showed evidence of prior infection with Jamestown Canyon virus, but none with California encephalitis.

Awareness of arboviruses in the United States has been heightened as a result of the recent outbreak of human illnesses caused by West Nile virus. That outbreak was originally thought to be due to St. Louis encephalitis (11). The case we describe is a further reminder that clinicians should consider several causative agents when a patient has a central nervous system infection, especially if mosquito exposure has occurred.

Further studies are needed to assess the risk for human infection by California encephalitis in coastal California and the role of various mosquito species in transmission.

### Acknowledgment

We thank William C. Reeves for his careful review of the manuscript.

Dr. Eldridge is Emeritus Professor of Entomology at the University of California, Davis, and continues to serve as director of the UC Mosquito Research Program. His area of specialization is the ecology of mosquitoes and mosquito-borne viral diseases of humans.

### References

1. Hammon WM, Reeves WC, Sather G. California encephalitis virus, a newly described agent. II. Isolations and attempts to identify and characterize the agent. J Immunol 1952;69:493-510.
2. Hammon WM, Reeves WC. California encephalitis virus, a newly described agent. I. Evidence of infection in man and other animals. California Medicine 1952;77:303-9.
3. Centers for Disease Control and Prevention. Summary of notifiable diseases, United States, 1998. MMWR Morb Mortal Wkly Rep 1998;47:22-5.
4. McJunkin JE, Khan RR, Tsai T. California-La Crosse encephalitis. Infect Dis Clin North Am 1998;47:83-93.
5. Campbell GL, Reeves WC, Hardy JL, Eldridge BF. Seroepidemiology of California and Bunyamwera serogroup viruses in humans in California. Am J Epidemiol 1992;136:308-19.
6. Campbell GL, Eldridge BF, Hardy JL, Reeves WC, Jessup DA, Presser SB. Prevalence of neutralizing antibodies against California and Bunyamwera serogroup viruses in deer from mountainous areas of California. Am J Trop Med Hyg 1989;40:428-37.
7. Lanzaro GC, Eldridge BF. A classical and population genetic description of two new sibling species of *Aedes* (*Ochlerotatus*) *increpitus* Dyar. Mosquito Systematics 1992;24:85-101.
8. Reeves WC, editor. Epidemiology and control of mosquito-borne arboviruses in California, 1943-1987. Sacramento: Calif Mosq Vector Control Assoc; 1990. p. 508.
9. Campbell GL, Reeves WC, Hardy JL, Eldridge BF. Distribution of neutralizing antibodies to California and Bunyamwera serogroup viruses in horses and rodents in California. Am J Trop Med Hyg 1990;42:282-90.
10. Fulhorst CF. The epidemiology and ecology of mosquito-borne viruses in coastal areas of California [dissertation]. Berkeley (CA): University of California, Berkeley; 1994.
11. Centers for Disease Control and Prevention. Outbreak of West Nile-like viral encephalitis—New York, 1999. MMWR Morb Mortal Wkly Rep 1999;48:845-9.

## References

[PDF_00165] Campbell G. L., Eldridge B. F., Hardy J. L., Reeves W. C., Jessup D. A., Presser S. B. (1989). Prevalence of neutralizing antibodies against California and Bunyamwera serogroup viruses in deer from mountainous areas of California.. Am J Trop Med Hyg.

[PDF_00175] Campbell G. L., Reeves W. C., Hardy J. L., Eldridge B. F. (1990). Distribution of neutralizing antibodies to California and Bunyamwera serogroup viruses in horses and rodents in California.. Am J Trop Med Hyg.

[PDF_00162] Campbell G. L., Reeves W. C., Hardy J. L., Eldridge B. F. (1992). Seroepidemiology of California and Bunyamwera serogroup bunyavirus infections in humans in California.. Am J Epidemiol.

[PDF_00182] Centers for Disease Control and Prevention (CDC) (1999). Outbreak of West Nile-like viral encephalitis--New York, 1999.. MMWR Morb Mortal Wkly Rep.

[PDF_00151] HAMMON W. M., REEVES W. C., SATHER G. (1952). California encephalitis virus, a newly described agent. II. Isolations and attempts to identify and characterize the agent.. J Immunol.

[PDF_00160] McJunkin J. E., Khan R. R., Tsai T. F. (1998). California-La Crosse encephalitis.. Infect Dis Clin North Am.

